# Prognostic Impact of Genetic Variants of MECP2 and TIRAP on Clinical Outcomes of Systemic Lupus Erythematosus with and without Nephritis

**DOI:** 10.3390/biom11091378

**Published:** 2021-09-18

**Authors:** Safaa I. Tayel, Nashwa M. Muharram, Dina S. Fotoh, Hany S. Elbarbary, Huda I. Abd-Elhafiz, Eman A. El-Masry, Ahmed E. Taha, Shimaa E. Soliman

**Affiliations:** 1 Medical Biochemistry and Molecular Biology Department, Faculty of Medicine, Menoufia University, Shebin el Kom 32511, Egypt; nashwa.mouharam@med.menofia.edu.eg (N.M.M.); Shaimaa_alshafaay@med.menofia.edu.eg (S.E.S.); 2 Medical Biochemistry Unit, College of Medicine, Al Baha University, Al Baha 65779, Saudi Arabia; 3 Physical Medicine, Rheumatology and Rehabilitation Department, Faculty of Medicine, Menoufia University, Shebin el Kom 32511, Egypt; dina.salem.12@med.menofia.edu.eg; 4 Renal Unit, Department of Internal Medicine, Faculty of Medicine, Menoufia University, Shebin el Kom 32511, Egypt; hany.elbarbari@med.menofia.edu.eg; 5 Renal Unit, Department of Internal Medicine, College of Medicine, King Faisal University, Al-Ahsa 31982, Saudi Arabia; 6 Clinical Pharmacology Department, Faculty of Medicine, Menoufia University, Shebin el Kom 32511, Egypt; Hoda.break@med.menofia.edu.eg; 7 Microbiology and Immunology Unit, Department of Pathology, College of Medicine, Jouf University, Sakaka 72388, Saudi Arabia; ealmasry@ju.edu.sa (E.A.E.-M.); aeattia@ju.edu.sa (A.E.T.); 8 Medical Microbiology and Immunology Department, Faculty of Medicine, Menoufia University, Shebin el Kom 32511, Egypt; 9 Medical Microbiology and Immunology Department, Faculty of Medicine, Mansoura University, Mansoura 35516, Egypt; 10 Medical Biochemistry Unit, Department of Pathology, College of Medicine, Qassim University, Buraydah 51452, Saudi Arabia

**Keywords:** arthritis, *MECP*, nephritis, systemic lupus erythematosus, *TIRAP*

## Abstract

Systemic lupus erythematosus (SLE) is a chronic autoimmune illness with a growing prevalence in many populations. Few studies have examined genetic predisposition to SLE, so we aimed to examine the clinical impact of the genetic polymorphisms *MECP2* rs2734647and *TIRAP* rs8177374 on the outcomes and therapeutic precision of SLE with and without nephritis. This study included 110 SLE patients—divided into 63 with lupus nephritis (LN), and 47 without nephritis—and 100 controls. Laboratory measurements including CRP, ESR, ACR, CBC, anti-ds-DNA, vitamin A, C3, and C4 were carried out, along with genotyping of *MECP2* rs2734647and *TIRAP* rs8177374 by real-time PCR and sequencing. Treg %, vitamin A, C3, and C4 were lower, whereas Th17 % was higher, in patients vs. controls (*p* < 0.001). The T allele of *MECP2* rs2734647 was higher in LN than in non-nephritis and control subjects. Moreover, the T allele of *TIRAP* rs8177374 was higher in LN than in non-nephritis and control subjects. The *MECP2* and *TIRAP* genes could play a role in predisposition to SLE, and can also predict disease progress to nephritis, helping to personalize medicine.

## 1. Introduction

Systemic lupus erythematosus (SLE) is a chronic autoimmune disease that causes the deposition of immune complexes and inflammatory cells in various bodily tissues, including the kidneys. Lupus nephritis (LN) is seen frequently in SLE, and is one of the most serious SLE complications, since it is the main forecaster of poor prognosis. The prevalence of LN differs depending on the studied population, being more common in people with Asian (55%), African (51%), and Hispanic (43%) ancestry compared with Caucasians (14%). Approximately 25% of LN patients still develop end-stage renal disease (ESRD) 10 years after the onset of renal affection [1,2].

Toll-like receptors (TLRs) are crucial originators of the immune response, both innate and acquired. Gene polymorphisms within TLRs trigger faults in vital TLR-related signaling routes, which subsequently raise the risk of autoimmune diseases [3].

The Toll-interleukin-1 receptor (TIR) domain-containing adaptor protein (TIRAP) signifies a fundamental intracellular signaling molecule controlling various immune responses. TIRAP is an adaptor protein that pairs myeloid differentiation factor 88 (MyD88) with TLRs to stimulate MyD88-dependent TLR signaling. The subsequent downstream signaling processes end in the triggering of diverse transcription factors including nuclear factor κB (NF-κB) and activated protein 1 (AP1). The instigation of TLR-mediated signaling pathways is crucial in the induction of proinflammatory cytokines by immune cells, and in monitoring host cell survival [4,5].

SNPs in the *TIRAP* gene have been linked to the initiation of, and susceptibility to, inflammatory diseases such as SLE [6]. *TIRAP* rs8177374 (C/T) SNP, which causes a leucine substitution at serine 180 of Mal (S180L), is associated with an increase in susceptibility to infectious diseases such as malaria, tuberculosis, and septic shock; moreover, S180L leads to an amino acid substitution in which Mal alters TLR2 and TLR4 signaling; hence, it could protect against life-threatening inflammatory disorders [7].

Improvements in immunosuppressive drugs and other pharmacological treatments have increased the mean 5-year survival for SLE patients. However, the lack of specificity, wide suppression of the immune cell functions, and effects on the replication of those cells frequently lead to serious toxicity and numerous adverse effects, so understanding the genetic polymorphism of different patients may provide a new insight into the molecular pathogenesis of LN and aid in the development of novel diagnostic and therapeutic tools [8].

Epigenetic mechanisms are heritable events that control gene expression by regulating the accessibility of DNA to the transcriptional complex [9]. Alterations of those mechanisms—including DNA methylation, histone modifications, and non-coding transcripts—are involved in the deregulation reported in many autoimmune/inflammatory diseases, including SLE [10].

One important gene, which contains the risk variant for autoimmune diseases as SLE according to numerous studies, is Methyl-CpG-binding protein 2 (MECP2)—a transcriptional regulator that controls the expression of methylation-sensitive genes. MECP2 recruits DNA methyltransferase 1 (DNMT1) during DNA methylation; it also recruits histone deacetylase complexes to the gene promoter, leading to chromatin conformation and, hence, silencing of gene expression [11].

Few studies have demonstrated the influence of methylation status on TLR expression in various illnesses, although one report declared that hypomethylation at gene promoters of TLR1, 2, 4, 6, 8, and 9 augments the expression of downstream genes, and treating Kawasaki disease patients with intravenous immunoglobulin can reduce TLRs’ mRNA expression and establish their methylation. Meanwhile, another report showed that CNS inflammation may be dysregulated by different means, where the TLR3, TLR9, RAGE, and MECP2 proteins could contribute [12,13].

Altered regulation of T-cell genes, which are sensitive to methylation, along with the fact that SLE is more common in women, makes the *MECP2* gene an important genetic factor of SLE [14]. The current study aimed to investigate the clinical impact of the genetic polymorphisms *MECP2* rs2734647and *TIRAP* rs8177374 on the clinical outcomes and selective management of SLE with or without nephritis.

## 2. Materials and Methods

This hospital-based case–control study was conducted on 110 Egyptian SLE patients recruited from the outpatient clinic of the Rheumatology, Physical Medicine, and Rehabilitation department, and the Nephrology clinic of the Internal Medicine Department, Menoufia University, between January and December 2020, in collaboration with the Medical Biochemistry and Molecular Biology, Medical Microbiology and Immunology, and Clinical Pharmacology departments. We classified 110 SLE patients based on evidence of clinical and laboratory characteristics of nephritis into two subgroups: 63 patients with LN, and 47 patients without nephritis. Moreover, 100 Egyptian healthy age- and sex-matched controls were also enrolled in the study. All patients and control subjects were females. The diagnosis of SLE was made as per the American College of Rheumatology (ACR) and the European League Against Rheumatism (EULAR)’s 2019 criteria for the classification of SLE [15,16]. The ACR/EULAR classification requires an antinuclear antibody (ANA) titer of at least 1:80 in HEp-2 cells, or an equivalent positive test, at least once. If that is present, 22 “additive-weighted” classification criteria are considered, comprising 7 clinical domains (constitutional, hematologic, neuropsychiatric, mucocutaneous, serosal, musculoskeletal, and renal) and 3 immunological domains (antiphospholipid antibodies, complement proteins, and SLE-specific antibodies). Each criterion is assigned a number of points, ranging from 2 to 10. Patients with at least one clinical criterion and 10 or more points are classified as having SLE. Laboratory tests indicated for SLE diagnosis include CBC with differential, serum creatinine, urinalysis with microscopy, ESR, CRP level, complement levels, liver function tests, spot protein/spot creatinine ratio, and autoantibody tests. Active disease is characterized by a systemic lupus erythematosus disease activity index (SLEDAI) score of more than 3 [17]. Laboratory tests for SLE disease activity include the following: antibodies to double-stranded DNA (dsDNA), complement (C3, C4) levels, erythrocyte sedimentation rate (ESR), and C-reactive protein (CRP).

Kidney biopsy was used to confirm the presence of LN, to aid in the classification of systemic lupus erythematosus (SLE) nephritis based on the International Society of Nephrology/Renal Pathology Society (ISN/RPS) classification, and to guide therapeutic decisions in the presence of the following features [18]: increased serum creatinine in the absence of strong evidence for another etiology; proteinuria of more than 1.0 g per 24 h, as confirmed by 24 h urine specimens or spot protein/spot creatinine ratios; proteinuria of 0.5 g or more per 24 h, along with either hematuria (≥5 RBCs/hpf) or cellular casts, as confirmed by a minimum of 2 tests within a short period. Additionally, in the absence of alternative causes, and during regular follow-up, laboratory abnormalities suggesting active lupus nephritis include hematuria or proteinuria. Laboratory tests to evaluate renal function in SLE patients with renal involvement included the following: blood urea nitrogen (BUN) testing, serum creatinine assessment, urinalysis (to check for protein, red blood cells (RBCs), and cellular casts) and spot urine tests for creatinine and protein concentration, and 24 h urine tests for creatinine clearance and protein excretion.

Patients with severe infection, immunodeficiency, malignancy, thyroid dysfunction, chronic liver disease, or chronic kidney disease from a different cause were excluded from our study. Male patients were excluded from the study due predominance of the disease in females—SLE is nearly 9 times more common in women than men over the life span [19]. In this regard, SLE appears to be one of the most sex-differentiated autoimmune diseases. Because SLE is most commonly diagnosed at reproductive age, the disease presents medical and psychosocial challenges that complicate family planning and pregnancy [20,21].

This study was conducted in accordance with the ethical standards of our institute and the 1964 Declaration of Helsinki. All participants gave informed consent before taking part in the study. This study was approved by the research ethical committee of the faculty of medicine, Menoufia University.

Full history and complete general and local joint examination were performed, along with pain evaluation using a visual analogue scale (VAS) [22]. Laboratory investigations—including complete blood count (CBC) using a Pentra-80 automated blood counter (ABX–Franc–Rue du Caducee-Paris Euromedecine-BP-7290.34184 Montpellier-Cedex4), and erythrocyte sedimentation rate (ESR)—were conducted. Renal function tests (serum creatinine and blood urea) were performed via colorimetric methods using DIAMOND diagnostics kits (Germany.) Albumin–creatinine ratio (ACR) and albuminuria were measured using a solid-phase enzyme-linked immunosorbent assay (ELISA) kit provided by DRG international Inc. (USA; cat no EIA-2361) from early morning spot urine collections and adjusted for urinary creatinine. Liver enzymes (alanine aminotransferase (ALT) and aspartate aminotransferase (AST)) were assayed via a kinetic UV-optimized IFCC method (LTEC Kit, England); CRP was measured via ELISA using a Sun Red Elisa Kit (China, catalog no. 201-12-1799). Antinuclear antibodies (ANAs) and human anti-double-stranded-DNA antibody (Anti-dsDNA) were measured using the MBS702970 and MBS269122 ELISA kits, respectively. Serum complement levels were measured using the Complement C3 ELISA Kit (ab108823) and Complement C4 ELISA Kit (ab108824). SLEDAI was used to assess the disease activity [18].

All SLE patients were followed up regularly to assess their renal functions and ACR. All SLE patients started their treatment systematically according to their clinical symptoms, usually with 200 mg of chloroquine phosphate twice daily, and corticosteroids for the treatment of skin manifestations and arthralgia. In case of insufficient clinical improvement, we added 50 mg of azathioprine twice per day with CBC follow up. Most patients with LN received 500 mg of mycophenolate mofetil, starting with two capsules per day, and potentially progressing up to six capsules per day after ACR follow-up. In advanced stages of LN, hospitalization, and administration of cyclophosphamide through IV infusion for 6 months was sometimes necessary.

Ten-milliliter blood samples were collected from each individual under aseptic conditions, and then divided into three parts: (a) 5 mL added to sterile tubes containing EDTA for complete blood count and CD markers, and DNA extraction for genotyping of *MECP2* rs2734647and *TIRAP* rs8177374 by real-time polymerase chain reaction (PCR); (b) 3.4 mL added to sterile plain tubes for assessment of vitamin A level, C-reactive protein, and serum creatinine; (c) 1.6 mL of blood delivered into a tube containing 0.4 mL trisodium citrate for erythrocyte sedimentation rate.

For genotyping of the *MECP2* rs2734647and *TIRAP* rs8177374 polymorphisms, DNA extractions were performed from peripheral blood utilizing a Gene JET Whole Blood Genomic DNA Purification Mini Extraction Kit (Thermo Scientific, EU/Lithuania). Genotyping of the C/T polymorphisms within the *MECP2* rs2734647 and *TIRAP* rs8177374 genes was carried out via real-time PCR and allelic discrimination assay utilizing a TaqMan probe (Applied Biosystems, USA). The primers, probes, and Master Mix (80×) were also supplied by Thermo Scientific. The used probe sequences for the *MECP2* rs2734647 gene were [VIC/FAM]:GTGGCGTTTCTGGGTGTCCCCTGTG [C/T] CTTTTGATATGGGAATACAGCATCA. The used probe sequences for the *TIRAP* rs8177374 gene were [VIC/FAM]: GAGGGCTGCACCATCCCCCTGCTGT[C/T] GGGCCTCAGCAGAGCTGCCTACCCA. Next, 1.5 μL of the primer/probe mixture was applied to a mixture of 10 μL of Master Mix and 3.5 μL of nuclease-free water. Five microliters of extracted DNA were added to each reaction. The cycling conditions were set as 10 min of preliminary denaturation at 95 °C, then 40 cycles of 15 s for denaturation at 94 °C, 60 s for primer annealing at 50 °C, and 2 min for extension at 72 °C, and finally 1 min at 72 °C as a terminal extension phase. Data were analyzed using the software accompanying the ABI7500 real-time PCR device V.2.0.1. Allele discrimination plots were constructed for *MECP2 and TIRAP*, and are shown in Supplementary Figure S1a,b, respectively.

### 2.1. Sequencing of the MECP2 rs2734647and TIRAP rs8177374 Genes

Genotyping of the *MECP2* rs2734647and *TIRAP* rs8177374 polymorphisms was further confirmed by sequencing. The Gene JET PCR Purification Kit (THERMO SCIENTIFIC, Lot 00424822, Lithuania) was used in the first step of DNA purification prior to the sequencing step. The primer sequences of the *MECP2* rs2734647 gene were F: AGCTTAAGCAAAGGAAATCTGG; R: GCTTTTCCCTGGGGATTG, while the primer sequences of the *TIRAP* rs8177374 gene were F: GTGTCTGGCCCTAATCTCATGAGGAAT; R: GCACTACACTCAGGAACACAGCAGAGTC. Afterwards, the samples were injected in cycler sequence after dilution with the primers to reach 10 pmol/uL, and the PCR product was diluted to reach 20 ng under the following thermal cycling conditions: 1 min at 96 °C, then 25 cycles of 10 s at 94 °C, 5 s for primer annealing at 61 °C, and 4 min for extension at 60 °C, followed by a pause at 4 °C, and then the CENTRI-SEP Kit (Princeton Separations, Adelphia, Lot: 12D7662) was used in the second purification step by applying heat shock to the samples with the Hi-Di reagent. The cycling conditions were 95 °C for 3 min followed by holding at 4 °C. Subsequently, samples were immediately stored at −20 °C for 2 min and frozen. The samples were analyzed using an AB Applied Biosystems HITACHI 3500 genetic analyzer. *MECP2* sequence analysis is demonstrated in Supplementary Figure S1c, whereas *TIRAP* sequence analysis is shown in Supplementary Figure S1d.

### 2.2. Statistical Analysis

IBM SPSS software package version 20.0 (IBM Corp., Armonk, NY, USA:) was used for data analysis. Data were evaluated by means of the Kolmogorov–Smirnov test to verify the normality of the distribution of variables. Quantitative data were analyzed as the mean and standard deviation (X ± SD). Qualitative data were set as number and percentage (No and %) and analyzed using the chi-squared test. The significance of the obtained results was decided at *p* < 0.05.

## 3. Results

Data from all 210 participants in the current study (110 SLE patients and 100 healthy controls) were analyzed statistically, and the results are shown in Table 1 and Table 2. The sample size was calculated to be 210 (110 cases and 100 controls), taking power at 80% and the significance level at 0.05, minor allele frequency in the control group of 5.1%, and an average odds ratio of 4.21. Age was matched between groups, where the mean age of the patients was (37.95 ± 9). Patients had a significant average visual analogue scale (VAS) of 30 (*p* < 0.001). Arthralgia was predominant in 63 (57.3%), while arthritis was detected in only 17 (15.5%), and 30 (27.35%) had no joint affection. Systolic blood pressure (SBP) was significantly different between groups (*p* < 0.001), while diastolic blood pressure (DBP) did not significantly vary (*p* = 0.221) (Table 1). Vitamin A was significantly lower in patients than in healthy controls (*p* < 0.001). Immune markers including regulatory T cells (Treg %) and complements three (C3) and four (C4) were markedly lower in patients than in controls (*p* < 0.001), whereas T helper 17 (Th17 %) was considerably elevated in patients compared to controls (*p* < 0.001). Markers of inflammation—including erythrocyte sedimentation rate (ESR) and C-reactive protein (CRP)—were demonstrably increased in patients (*p* < 0.001), indicating the presence of active disease. Elements of CBC—including Hb and WBCs—were significantly higher in patients (*p* < 0.001), while platelets did not vary significantly between groups (*p* = 0.076). Renal functions were elevated in patients, indicating kidney involvement, where serum urea (*p* = 0.001), serum creatinine (*p* < 0.001), and albumin–creatinine ratio (ACR) (*p* < 0.001) were all markedly elevated in patients compared to controls. The mean SLEDAI in patients was (6.4 ± 2.8), proving disease activity, while the mean anti-ds-DNA titer was relatively high (88.23 ± 60.71). Treatment of SLE varied between patients, where all patients (100%) received azathioprine, 47 patients (42.7%) were given chloroquine phosphate and corticosteroids, while mycophenolate mofetil was given to 27 (24.5%), and only 10 (9.1%) patients were treated with cyclophosphamide (Table 1).

The genotype and allele distribution of MECP2 rs2734647 and TIRAP rs8177374 were investigated, and the Hardy–Weinberg equilibrium was analyzed. Neither SNP deviated from the Hardy–Weinberg equilibrium, where MECP2 rs2734647 was 0.052 in patients and 0.067 in controls, while TIRAP rs8177374 was 0.626 in patients and 0.266 in controls.

The presence of the T allele of MECP2 rs2734647 increases susceptibility to the disease where the individual CT and TT genotypes increase the risk of SLE with an odds ratio (OR) of 2.648 (*p* = 0.005) and 4.569 (*p* = 0.024), respectively, while having both (CT and TT) increased the risk with an OR of 2.951 (*p* = 0.001). T allele frequency was 25.5% in patients, which was markedly higher than controls (11%), with an OR of 2.763 (*p* < 0.001). Moreover, we noticed that the CT and TT genotypes of TIRAP rs8177374 increased the risk of SLE with an OR of 3.283 (*p* < 0.001) and 3.203 (*p* = 0.172), respectively, while having both (CT and TT) increased the risk with an OR of 3.274 (*p* < 0.001). T allele frequency was 23.2% in patients; however, in controls, it was only 10%, with an OR of 2.716 (*p* < 0.001) (Table 2).

We further classified 110 SLE patients based on renal affection into SLE with nephritis (*n* = 63) and SLE without nephritis (*n* = 47), and compared both subgroups with controls, as shown in Table 3 and Table 4. Age was matched between groups (*p* = 0.094). Occupation (*p* = 0.001) and menstrual history (*p* = 0.002) were significantly different between groups, where workers represented 41.3% of the nephritis group but only 10.6% of the non-nephritis group. Moreover, 82.5% of nephritis patients had regular menstruation, compared to 66% in the non-nephritis group and 56% of controls. Furthermore, VAS was markedly elevated in the nephritis group, with an average of 30, while in the non-nephritis group it was 20 and in controls it was 0 (*p* < 0.001), showing significant differences between groups. Concerning affected joints, arthralgia was evident in 54% of the nephritis group, while being a more common manifestation in the non-nephritis group (61.7%) (*p* < 0.001), and arthritis was more prevalent in the nephritis group (19%) than in the non-nephritis group (10.6%) (*p* < 0.001). Systolic (*p* < 0.001) and diastolic BP (*p* = 0.019) were significantly different between groups; in addition, both were higher in the nephritis than the non-nephritis group (*p* = 0.029).

Vitamin A was noticeably lower in the nephritis group than in the non-nephritis and control groups (*p* < 0.001). The SLEDAI was distinctly higher in nephritis patients than in non-nephritis patients (*p* < 0.001), demonstrating more active disease in the former, although the anti-ds-DNA titer was to some extent equal in both groups (*p* = 0.508). Standard management included azathioprine, which was given to all patients; cyclophosphamide and mycophenolate mofetil were given to nephritis patients only (*p* = 0.005, *p* < 0.001, respectively), while chloroquine phosphate was taken by 41.3% of nephritis patients and 44.7% of non-nephritis patients (*p* = 0.721) (Table 3).

The immune markers Treg percentage, C3, and C4 were definitely lower in nephritis patients compared to non-nephritis patients (*p* < 0.001) and controls (*p* < 0.001). Conversely, Th17 percentage was clearly increased in nephritis patients compared with non-nephritis patients (*p* < 0.001) and controls (*p* < 0.001). Moreover, inflammatory markers including ESR and CRP were elevated in both nephritis (*p* < 0.001) and non-nephritis patients (*p* < 0.001 and *p* = 0.002, respectively) versus controls. Parameters of renal functions—including serum creatinine, serum urea, and ACR—were all prominently elevated in nephritis patients compared with non-nephritis patients (*p* < 0.001) and controls (*p* < 0.001), and the differences between non-nephritis patients and controls were not significant (Table 3).

*MECP2* rs2734647 and *TIRAP* rs8177374 frequencies and dissemination were analyzed among subgroups, as shown in Table 4. The T allele frequency of *MECP2* rs2734647 was significantly higher in nephritis patients (33.3%) than in non-nephritis patients (14.9%, OR1 = 2.857, *p* = 0.002) or controls (11%, OR2 = 4.045, *p* < 0.001), although the T allele difference between non-nephritis patients and controls was not significant (OR3 = 1.416, *p* = 0.344).

Furthermore, CT + TT of *MECP2* was prominently elevated in nephritis patients (50.8%) compared to non-nephritis patients (27.7%, OR1 = 2.70, *p* = 0.016) and controls (19%, OR2 = 4.401, *p* < 0.001), and no significant difference was noted between non-nephritis patients and controls (OR3 = 1.63, *p* = 0.238). Concerning *TIRAP* rs8177374, the T allele was prominently higher in nephritis patients (30.2%) than in non-nephritis patients (13.8%, OR1= 2.691, *p* = 0.005) and controls (10%, OR2 = 3.886, *p* < 0.001), although the T allele difference between non-nephritis patients and controls was not significant (OR3 = 1.444, *p* = 0.334).

Moreover, CT + TT of *TIRAP* was noticeably higher in nephritis patients (54.0%) than in non-nephritis patients (25.5%, OR1 = 3.420, *p* = 0.003) and controls (18%, OR2 = 5.341, *p* < 0.001), and no significant distinction was observed between non-nephritis patients and controls (OR3 = 1.562, *p* = 0.293) (Table 4).

**Figure 1:**
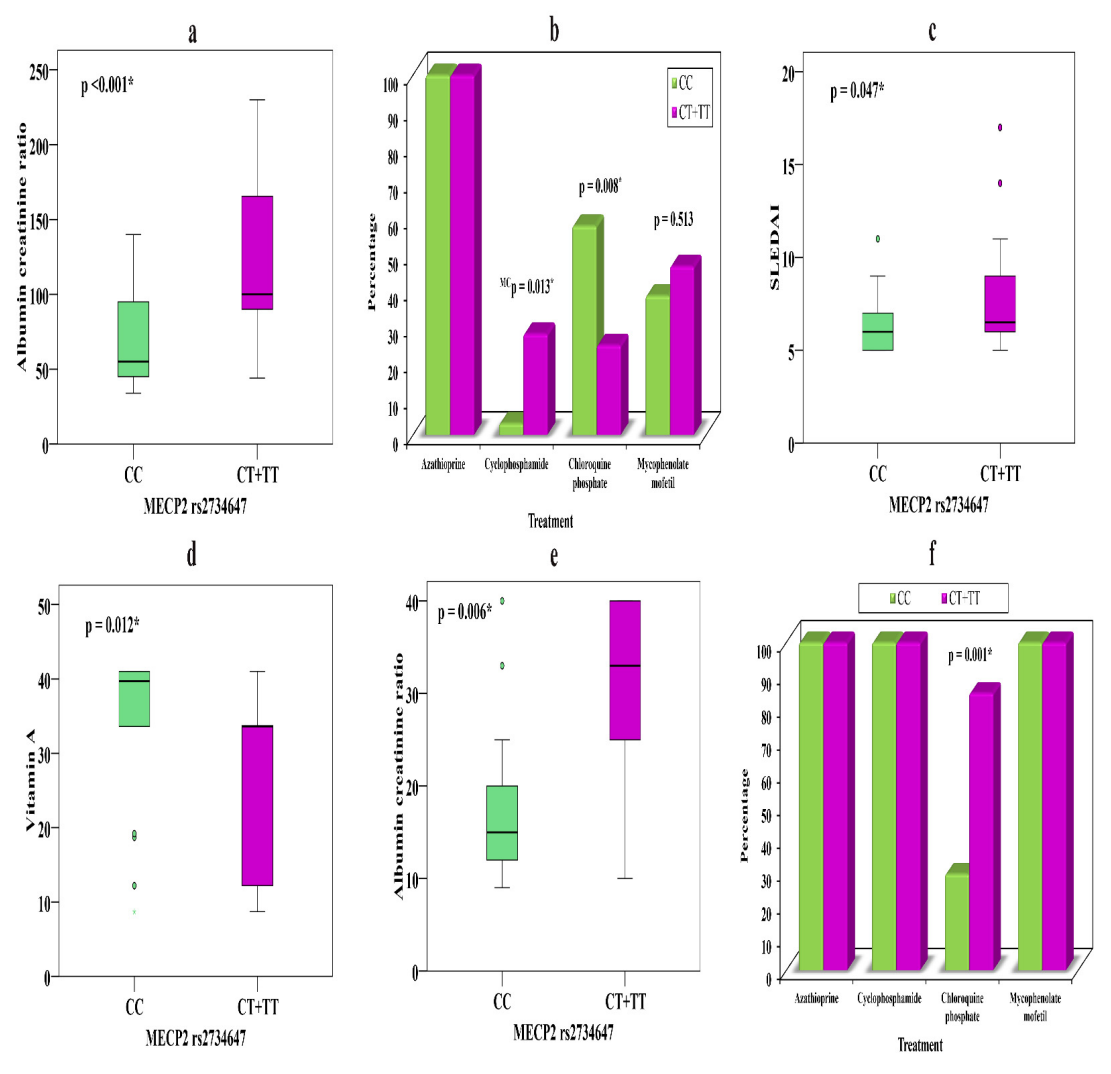
(**a**) Relationship between *MECP2* rs2734647 and ACR in LN. (**b**) Relationship between *MECP2* rs2734647 and treatment in LN. (**c**) Relationship between *MECP2* rs2734647 and SLEDAI in LN. (**d**) Relationship between *MECP2* rs2734647 and vitamin A in patients without nephritis. (**e**) Relationship between *MECP2* rs2734647 and ACR in patients without nephritis. (**f**) Relationship between *MECP2* rs2734647 and treatment in patients without nephritis. *—significant.

The relationships of *MECP2* rs2734647 genotypes CC (*n* = 31) and CT + TT (*n* = 32) with various parameters in SLE patients with nephritis are presented in Table 5. We observed that presence of the T allele in CT + TT was significantly associated with a higher ACR (*p* < 0.001) (Figure 1a), which might explain its association with increased risk of nephritis. Moreover, carriers of the T allele might show appropriate responses to cyclophosphamide, as nine patients (28.1%) took this drug, while there was only one (3.2%) carrier of the C allele in CC (*p* = 0.013), which might indicate that the C allele can precipitate resistance to cyclophosphamide.

Conversely, carriers of the C allele showed a conventional response to chloroquine phosphate 18 (58.1%) that was not evident in T allele carriers 8 (25%) (*p* = 0.008) (Figure 1b), which may prove that genetic background can determine the drug of choice in individual therapy. Carriers of the T allele showed higher SLEDAI scores (*p* = 0.047) than those carrying the C allele (Figure 1c), which might explain a severe form of the disease (Table 5).

The relationships of *MECP2* rs2734647 genotypes CC (*n* = 34) and CT + TT (*n* = 13) with various parameters in SLE patients without nephritis show that carriers of CT + TT have noticeably lower vitamin A levels (*p* = 0.012) (Figure 1d) and elevated ACR (*p* = 0.006) (Figure 1e) and showed better drug response to chloroquine phosphate 11 (84.6%) than C allele carriers 10 (29.4%) (*p* = 0.001) (Figure 1f).

**Figure 2:**
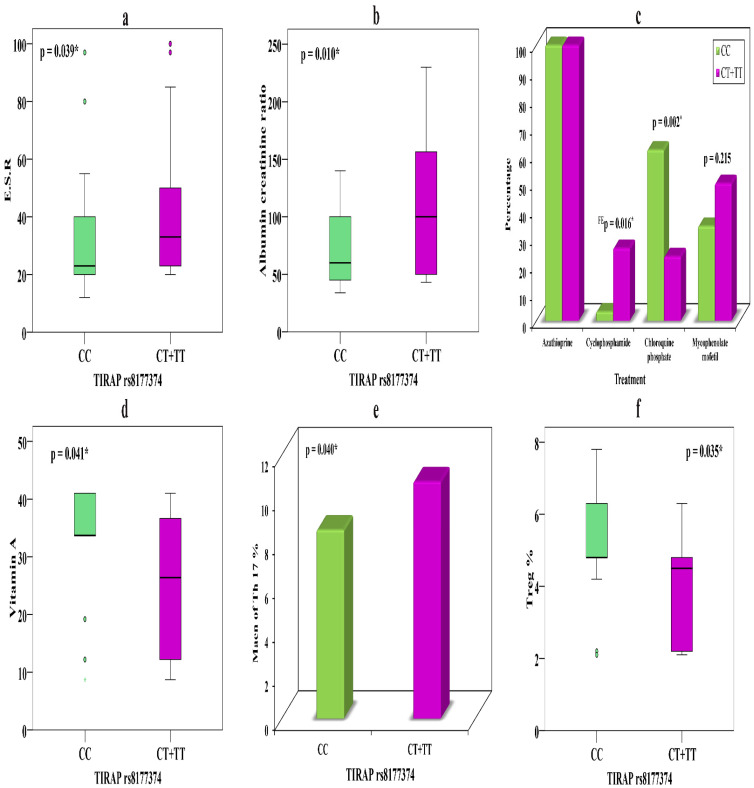
(**a**) Relationship between *TIRAP* rs8177374 and ESR in LN. (**b**) Relationship between *TIRAP* rs8177374 and ACR in LN. (**c**) Relationship between *TIRAP* rs8177374 and treatment in LN. (**d**) Relationship between *TIRAP* rs8177374 and vitamin A in patients without nephritis. (**e**) Relationship between *TIRAP* rs8177374 and Th17 % in patients without nephritis. (**f**) Relationship between *TIRAP* rs8177374 and Treg % in patients without nephritis. *—significant.

The relationships between *TIRAP* rs8177374 genotypes CC (*n* = 29) and CT + TT (*n* = 34) and various parameters in SLE patients with nephritis is presented in Table 6. Carriers of the T allele in CT + TT show noticeably higher ESR (*p* = 0.039) (Figure 2a) and ACR (*p* = 0.01) (Figure 2b) than those carrying the C allele which might illuminate its association with nephritis.

Carriers of the T allele showed evident response to cyclophosphamide in nine (26.5%) patients, while only one (3.4%) C allele carrier took cyclophosphamide (*p* = 0.016). However, chloroquine phosphate showed divergent results, with 18 (62.1%) C allele carriers showing a therapeutic response, while only 8 (23.5%) T allele carriers did so, suggesting that the latter might cause drug resistance (*p* = 0.002) (Figure 2c) (Table 6).

Concerning the relationships of *TIRAP* rs8177374 genotypes CC (*n* = 35) and CT + TT (*n* = 12), we observed that carriers of CT + TT showed markedly lower vitamin A levels (*p* = 0.041) (Figure 2d) and an elevated Th17 percentage (*p* = 0.04) (Figure 2e), along with a distinctly lower Treg percentage (*p* = 0.035) (Figure 2f), compared with C allele carriers. These significant relationships with immune markers might feature their association with disease risk.

Following adjustment for age, SBP, DBP, and arthralgia and arthritis patterns, univariate, and multivariate regression analysis of all SLE patients relative to controls revealed that the *MECP2* rs2734647 genotypes CT and TT could be independent predictor of disease, with (OR 2.951, *p* = 0.001) and (OR 4.343, *p* = 0.015), respectively.

Moreover, *TIRAP* rs8177374 genotypes CT and TT could also predict the disease, with (OR 3.274, *p* < 0.001) and (OR 6.540, *p* = 0.005), respectively.

Univariate and multivariate regression analysis for SLE patients with and without nephritis revealed that the *MECP2* rs2734647 genotypes CT and TT are independent predictors of nephritis in SLE patients, with (OR 2.70, *p* = 0.016) and (OR 2.648, *p* = 0.034), respectively.

Moreover, the *TIRAP* rs8177374 genotypes CT and TT could also predict nephritis in SLE, with (OR 3.420, *p* = 0.003) and (OR 3.942, *p* = 0.006), respectively.

## 4. Discussion

Systemic lupus erythematosus (SLE) is a clinicopathologically diverse chronic autoimmune disease that causes distress to various tissues and organs [23]. In Egypt, 89.7% of SLE patients are females, while only 10.3% are males. The mutual cumulative displays include arthritis, malar rash, leukopenia, photosensitivity, nephritis, and neuropsychiatric lupus [24].

Genetic, epigenetic, and environmental factors might play crucial roles in the advance of SLE [25]. Recurrent flares and sustained phases of active illness are concomitant with adverse outcomes in lupus patients, and cause damage to tissues and organs [26]. LN is a dangerous outcome of SLE, adding considerably to SLE-related morbidity and mortality [27]. Early recognition and management of LN are imperative to diminish the risk of inflammation-prompted permanent kidney damage, and to reserve renal function. Additionally, the study of pathway-specific immune deregulation can enable accurate, personalized pharmacotherapy for LN [28].

We aimed in the present study to evaluate the connection between the *MECP2* rs2734647 and *TIRAP* rs8177374 variants and SLE risk, and to establish their relationships with immune markers and medical management, in addition to disease progression and activity. Moreover, we decided to resolve their relationship with LN and their impact on kidney functions. We found that the T allele of *MECP2* rs2734647 increases the risk of SLE. Additionally, we noticed that the T allele of *TIRAP* rs8177374 also increases disease risk. These striking findings may prove both genes’ association with SLE susceptibility. MECP2 recruits the histone deacetylase to the promoter regions of goal genes that provoke heterochromatin construction and transcriptional embarrassment [29]; additionally, it can control gene expression via DNMT1 recruitment. *MECP2* reduces the release of IFN-γ by Th cells, producing a partial immune inhibition [30]. Previous studies revealed a possible genetic association of *MECP2* single-nucleotide polymorphisms (SNPs) with susceptibility to SLE [23,29,31,32,33,34]; moreover, MECP2 rs2734647 is related to other diseases, such as Rett syndrome [35], schizophrenia [36], and aggressive social behavior [37].

*MECP2* is reflected as a gene related to SLE, attributed to its crucial function in the transcriptional suppression of genes that elude methylation and are highly expressed in SLE, and owing to the distorted control of T-cell genes, which are sensitive to methylation [14,29,38].

Doudar et al. [31] found that the A allele of *MECP2* rs1734791 is the risk allele for lupus in Egyptian populations. Rzeszotarska et al. [34] stated that rs2075596, rs1734787, rs17435, and rs2239464 within the *MECP2* gene are more common in SLE patients than in healthy controls and could possibly constitute predictive elements for the progression and course of SLE in Polish populations. Alesaeidi et al. [39] found that in Iranian patients, rs1734787 and rs1734791 of *MECP2* were correlated with SLE progression.

Studies on the relationship of *TIRAP* rs8177374 (C/T) polymorphism with SLE are limited; thus, we found a remarkable association with SLE susceptibility. *TIRAP* rs8177374, which codes a leucine replacement at serine 180 of Mal (S180L), was found to shield against pneumococcal disease, bacteremia, malaria, tuberculosis, and SLE [6,40]. Rupasree et al. [41] declared that the TLR4, TLR9, and TIRAP polymorphisms have an association with serological and phenotypic subsets of SLE via changes in MHC2TA and HLA-DR expression. Loss of TIRAP expression in SLE creates debate on the variants of significance in many populations, as is the case for many immunogenetic variants [5].

Few studies have inspected the relationship between *MECP2 and TIRAP* polymorphisms and LN, so we intended to examine their correlation with nephritis. We found that the T alleles of *MECP2* rs2734647 and *TIRAP* rs8177374 were considerably higher in LN patients than in non-nephritis patients and controls; however, the T allele difference between non-nephritis patients and controls was not significant. Concerning the relationships of the studied SNPs with clinical features of SLE, CT + TT of *MECP2* rs2734647 was correlated with elevated ACR, higher SLEDAI, and therapeutic drugs, while CT + TT of *TIRAP* rs8177374 was correlated with elevated ACR, higher ESR, and therapeutic drugs in nephritis patients, which may contribute to disease severity and progression, and could be linked with SLE prognosis. In non-nephritis patients, CT + TT of *MECP2* was associated with lower vitamin A and elevated ACR and showed a better response to chloroquine phosphate than CC carriers, yet CT + TT of *TIRAP* showed noticeably lower vitamin A and Treg % and elevated Th17 % relative to CC carriers.

Bentham et al. [42] announced in their GWAS analysis that *MECP2* rs1734787 is an SLE risk factor for people of European ancestry. Rzeszotarska et al. [34] found that variants within the *MECP2* gene (rs1734787 and rs2239464) might be related to earlier disease inception and more rapid disease passage and concluded that variants in the *MECP2* and CCR5 genes might have an impact on elevated ALT and AST values. Conversely, Doudar et al. [31] did not detect a significant relationship between rs1734791 in *MECP2* and disease severity or activity. Additionally, Alesaeidi et al. [39] demonstrated no significant connection between rs1734791 or rs1734787 in *MECP2* and clinical features of SLE.

Rupasree et al. [41] declared that *TIRAP* S180L showed a positive association with alopecia and malar rashes, and an inverse relation with psychosis. TLR T399I and *TIRAP* S180L showed positive associations with anti-Ro, demonstrating the influence of TLR and *TIRAP* genotypes on specific autoantibody formation. Endogenous nuclear particles experiencing receptor-mediated endocytosis can extend endosomes and interact with endosomal TLRs. Genetic variants of TLR3 (dsRNA), TLR7/8 (ssRNA), and TLR9 (DNA) can precipitate LN. Activation of TLR3 on antigen-presenting cells (APCs) or renal mesangial cells can aggravate LN by enhancing the expression of CXCL1/GROα to recruit PMNs to the inflammation area, contributing to renal damage [43,44].

Autoantibodies to double-stranded DNA (dsDNA), and markers of complement activation such as C3 and C4, are widely utilized in clinical practice in the diagnosis and surveillance of patients with LN, and these LN biomarkers were evidently used in this study. TIRAP is implicated in the TLR2- and TLR4-mediated MyD88-dependent signaling pathways; thus, *TIRAP* gene variants can modulate the signaling pathways and further the secretion of cytokines and inflammatory mediators, which can influence the course and progression of SLE. Chloroquine phosphate causes endosomal TLR blockade, which modulates the inflammatory process and cytokine production, but because of its serious side effects, the dose that we used in the clinic is not effective in all cases [45]. A previous animal study postulated that the administration of mycophenolate mofetil could inhibit TLR4 expression and signaling on the surface of monocytes, thus hindering the monocytes’ immune function. Therefore, the immune markers were downregulated with the administration of mycophenolate mofetil, due to inhibition of cytokines and overproduction of chemokines, and could protect the renal tissues [46]. Cyclophosphamide is an alkylating agent that is used in severe cases of LN to suppress the immune system by diminishing the overproduction and infiltration of neutrophils [47], as well as downregulation of TLR2, which activates cytokines such as IL-10 by differentiating the Treg cells (TLR2-expressing dependent cell), which are the most important sources of IL-10 [48].

Many known LN susceptibility genes have functions that mediate inflammation via cytokine production and activation of leukocytes. The known cellular pathways mediated by these variant gene products also provide valuable mechanistic insights for the development of personalized therapeutics. Defining genetic variants that reliably predict disease susceptibility, risk of progression, and response to treatment carries tremendous potential to improve and personalize patient care [49].

Finally, a genome-wide association (GWA) study in Koreans revealed 10 risk regions—STAT1-STAT4, TNFSF4, TNFAIP3, IKZF1, HIP1, IRF5, BLK, WDFY4, ETS1, and IRAK1-MECP2—to be implicated in SLE [50].

The limitations of this study include its small sample size; thus, we recommend a wide-scale study in multiple centers to validate our results. If proved, these genetic tests could be used in the routine lab workup of SLE patients to predict their risk of LN, and whether or not they need extensive follow-up. Moreover, it would be better to conduct a long-term follow-up study to investigate the possible correlation between outcomes of specific treatments and drugs used, to validate the use of personalized medicine in SLE. Furthermore, we encourage further studies to establish correlations between *MECP* and *TIRAP* variants, with their relative gene expression levels, in order to investigate possible relationships with SLE pathogenesis and outcomes.

## 5. Conclusions

The genetic loci within the *MECP2* and *TIRAP* genes could play a role in predisposition to SLE and can also predict disease onset and severity. The association of TIRAP polymorphism with lack of Treg percentage, decline in vitamin A, and rise in Th17percentage can facilitate SLE pathology. Moreover, the association of MECP2 and TIRAP with ACR and SELDAI can influence SLE activity and progress to lupus nephritis. We recommend further investigations regarding TLRs and their signaling pathways in LN that might identify a therapeutic target and permit accurate personalized therapy in LN.

## Figures and Tables

**Table 1:** Comparison between the two studied groups according to different parameters.

Variables	Mean/Median	Patients (*n* = 110)	Control (*n* = 100)	OR 95% C.I (LL–UL)	*p*
**Age (years)**	Mean ± SD.	37.95 ± 9	36.8 ± 9.5	1.013 (0.98–1.04)	0.374 *^t^*
**Occupation**	Housewife	79 (71.8%)	64 (64%)		0.226 ^χ2^
	Worker	31 (28.2%)	36 (36%)	0.698 (0.39–1.25)	
**Menstrual history**	Irregular	27 (24.5%)	44 (44%)		0.003 *^,χ2^
	Regular	83 (75.5%)	56 (56%)	2.415 (1.34–4.34)	
**VAS**	Median (Min.–Max.)	30 (10–90)	0 (0–20)	1.577 (1.35–1.84)	<0.001 *^,U^
**Arthralgia**		63 (57.3%)	2 (2%)	65.681 (15.406–280.02)	<0.001 *^,χ2^
**Arthritis**		17 (15.5%)	0 (0%)	–	0.988 ^χ2^
**Normal**		30 (27.3%)	98 (98%)	0.008 (0.002–0.033)	<0.001 *^,χ2^
**Pulse (/min)**	Median (Min.–Max.)	75 (65–80)	65 (65–80)	1.222 (1.16–1.29)	<0.001 *^,U^
**SBP mm Hg**	Mean ± SD.	112.5 ± 14.5	121.6 ± 13.5	0.957 (0.94–0.98)	<0.001 *^,^*^t^*
**DBP mm Hg**	Mean ± SD.	76.2 ± 7.3	77.2 ± 4.5	0.973 (0.93–1.02)	0.231 *^t^*
**Vitamin A (µg/L)**	Median (Min.–Max.)	19.2 (8.7–41)	51.7 (33.3–62.5)	0.647 (0.54–0.77)	<0.001 *^,U^
**Th 17 %**	Mean ± SD.	10.7 ± 3.2	3.3 ± 0.59	–	0.963 *^t^*
**T Reg %**	Median (Min.–Max.)	3.5 (1.3–7.8)	7.1 (5.7–8.9)	0.039 (0.01–0.16)	<0.001 *^,U^
**C3**	Mean ± SD.	66.8 ± 15.6	87 ± 4.1	0.843 (0.80–0.89)	<0.001 *^,^*^t^*
**C4**	Median (Min.–Max.)	10 (8–25)	15 (12–25)	0.825 (0.77–0.88)	<0.001 *^,U^
**E.S.R (mL/h)**	Median (Min.–Max.)	31 (12–100)	11 (5–33)	1.445 (1.30–1.61)	<0.001 *^,U^
**ALT (U/L)**	Median (Min.–Max.)	15 (8–85)	15.5 (15–20)	1.016 (0.98–1.06)	0.434 ^U^
**AST (U/L)**	Median (Min.–Max.)	17 (12–70)	19.5 (17–22)	1.025 (0.98–1.06)	0.301 ^U^
**HB (g/dL)**	Mean ± SD.	11.2 ± 1.7	9.7 ± 0.50	3.710 (2.64–5.58)	<0.001 *^,^*^t^*
**Platelets (×10^3^)**	Median (Min.–Max.)	276 (175–385)	236 (175–297)	1.006 (1.0–1.01)	0.009 *^,U^
**WBC (×10^3^)**	Median (Min.–Max.)	4.6 (3.4–9.6)	4 (3.4–4.5)	3.918 (2.39–6.43)	<0.001 *^,U^
**C.R.P**	Median (Min.–Max.)	12 (4–34)	10 (5–20)	1.200 (1.12–1.29)	<0.001 *^,U^
**Creatinine (mg/dL)**	Median (Min.–Max.)	1.1 (0.1–6.7)	0.9 (0.6–1)	25.855 (5.74–116.49)	<0.001 *^,U^
**Urea (mg/dL)**	Median (Min.–Max.)	30 (15–150)	34 (27–36)	1.015 (0.99–1.04)	0.162 ^U^
**Albumin–creatinine ratio (mg/gm)**	Median (Min.–Max.)	44.5 (9–230)	16.5 (4–28)	1.114 (1.07–1.16)	<0.001 *^,U^
**Treatment**	Azathioprine	110 (100%)	–	–	–
	Cyclophosphamide	10 (9.1%)	–	–	–
	Chloroquine phosphate	47 (42.7%)	–	–	–
	Mycophenolate mofetil	27 (24.5%)	–	–	–
**SLEDAI**	Mean ± SD.	6.4 ± 2.8	–	–	–
	Median (Min.–Max.)	6 (0–17)	–	–	–
**Anti-ds-DNA titer**	Mean ± SD.	88.23 ± 60.71	–	–	–
	Median (Min.–Max.)	100 (9–170)	–	–	–

^χ^2^^: Chi-squared test; ^*t*^: Student’s *t*-test; ^U^: Mann–Whitney test; *p*: *p*-value for comparing between the studied groups; *: statistically significant at *p* ≤ 0.05. Visual analogue scale (VAS); erythrocyte sedimentation rate (ESR); albumin–creatinine ratio (ACR); alanine aminotransferase (ALT); and aspartate aminotransferase (AST); C-reactive protein (highly sensitive) (CRP); anti-double-stranded-DNA titer (anti-ds-DNA); and complement C3 and C4 systemic lupus erythematosus disease activity index (SLEDAI).

**Table 2:** Comparison between the two studied groups according to *MECP2* rs2734647 and *TIRAP* rs8177374.

Gene	Patients (*n* = 110)	Control (*n* = 100)	OR (95% C.I) (LL–UL)	*p*
***MECP2* rs2734647**				
**CC**	65 (59.1%)	81 (81%)	1.000	
**CT**	34 (30.9%)	16 (16%)	2.648 * (1.34–5.22)	0.005 *
**TT**	11 (10%)	3 (3%)	4.569 (1.22–17.06)	0.024 *
**(CT + TT)**	45 (40.9%)	19 (19%)	2.951 (1.58–5.53)	0.001 *
**HWE**	0.052	0.067		
**Allele**				
**C**	164 (74.5%)	178 (89%)	1.000	
**T**	56 (25.5%)	22 (11%)	2.763 (1.615–4.726)	<0.001 *
***TIRAP* rs8177374**				
**CC**	64 (58.2%)	82 (82%)	1.000	
**CT**	41 (37.3%)	16 (16%)	3.283 (1.690–6.377)	<0.001 *
**TT**	5 (4.5%)	2 (2%)	3.203 (0.602–17.051)	0.172
**(CT + TT)**	45 (40.9%)	18 (18%)	3.274 (1.73–6.18)	<0.001 *
**HWE**	0.626	0.266		
**Allele**				
**C**	169 (76.8%)	180 (90%)	1.000	
**T**	51 (23.2%)	20 (10%)	2.716 * (1.554–4.746)	<0.001 *

OR: odds ratio; LL: lower limit; UL: upper limit; C.I: confidence interval; *: statistically significant at *p* ≤ 0.05; HWE: Hardy–Weinberg equilibrium.

**Table 3:** Comparisons between the three studied groups according to different parameters.

Variables	SLE Patients (*n* = 110)		Control (*n* = 100)	Test of Sig.	*p*
	With Nephritis (*n* = 63)	Without Nephritis (*n* = 47)			
**Age (years) Mean ± SD.**	36.4 ± 8.9	40 ± 8.8	36.8 ± 9.5	F = 2.395	0.094
**Occupation**					
**Housewife**	37 (58.7%)	42 (89.4%)	64 (64%)	χ^2^ = 13.099 *	0.001 *
**Worker**	26 (41.3%)	5 (10.6%)	36 (36%)		
**Menstrual History**					
**Irregular**	11 (17.5%)	16 (34%)	44 (44%)	χ^2^ = 12.166 *	0.002 *
**Regular**	52 (82.5%)	31 (66%)	56 (56%)		
**VAS Median (Min.–Max.)**	30 (10–90)	20 (10–70)	0 (0–20)		
**Sig. bet. grps.**	*p*_1_ = 0.048 *, *p*_2_ < 0.001 *, *p*_3_ = 0.001 *				
**Arthralgia**	34 (54%)	29 (61.7%)	2 (2%)	χ^2^ = 75.631 *	<0.001 *
**Arthritis**	12 (19%)	5 (10.6%)	0 (0%)	χ^2^ = 19.374 *	<0.001 *
**Normal**	17 (27%)	13 (27.7%)	98 (98%)	χ^2^ = 110.099 *	<0.001 *
**Pulse Median (Min.–Max.)**	75 (65–80)	80 (65–80)	65 (65–80)	K = 52.130 *	<0.001 *
**Sig. bet. grps.**	*p*_1_ = 0.110, *p*_2_ < 0.001 *, *p*_3_ = 0.001 *				
**Systolic BP mmHg**					
**Mean ± SD.**	115.4 ± 16.2	108.5 ± 10.8	121.6 ± 13.5	F = 14.674 *	<0.001 *
**Sig. bet. grps.**	*p*_1_ = 0.029 *, *p*_2_ = 0.016 *, *p*_3_ < 0.001 *				
**Diastolic BP mmHg**					
**Mean ± SD.**	77.5 ± 8.4	74.5 ± 5	77.2 ± 4.5	F = 4.037 *	0.019 *
**Sig. bet. grps.**	*p*_1_ = 0.029 *, *p*_2_ = 0.961, *p*_3_ = 0.030 *				
**Vitamin A (µg/L)**				K = 155.791 *	<0.001 *
**Median (Min.–Max.)**	17.8 (8.7–41)	33.7 (8.7–41)	51.7 (33.3–62.5)		
**Sig. bet. grps.**	*p*_1_ = 0.004 *, *p*_2_ < 0.001 *, *p*_3_ < 0.001 *				
**Treatment**					
**Azathioprine**	63 (100%)	47 (100%)	–	-	-
**Cyclophosphamide**	10 (15.9%)	0 (0%)	–	χ^2^ = 8.206	^FE^*p* = 0.005 *
**Chloroquine phosphate**	26 (41.3%)	21 (44.7%)	–	χ^2^ = 0.128	0.721
**Mycophenolate mofetil**	27 (42.9%)	0 (0%)	–	χ^2^ = 26.695	<0.001 *
**SLEDAI** **Median (Min.–Max.)**	6 (5–17)	5 (0–11)	–		
**Anti-ds-DNA** **titer Median (Min.–Max.)**	100 (9–170)	100 (9–170)	–		
**Th17 %**					
**Mean ± SD.**	11.8 ± 2.7	9.2 ± 3.2	3.3 ± 0.59	F = 322.570 *	<0.001 *
**Sig. bet. grps.**	*p*_1_ < 0.001 *, *p*_2_ < 0.001 *, *p*_3_ <0.001 *				
**Treg %**					
**Median (Min.–Max.)**	2.8 (1.3–6.3)	4.8 (2.1–7.8/)	7.1 (5.7–8.9)	K = 152.721 *	<0.001 *
**Sig. bet. grps.**	*p*_1_ < 0.001 *, *p*_2_ < 0.001 *, *p*_3_ = 0.445				
**C3**					
**Mean ± SD.**	58.3 ± 10.3	78.1 ± 14.3	87 ± 4.1	F = 187.862 *	<0.001 *
**Sig. bet. grps.**	*p*_1_ < 0.001 *, *p*_2_ < 0.001 *, *p*_3_ < 0.001 *				
**C4**				K = 104.050 *	<0.001 *
**Median (Min.–Max.)**	9 (8–20)	16 (9–25)	15 (12–25)		
**Sig. bet. grps.**	*p*_1_ < 0.001 *, *p*_2_ < 0.001 *, *p*_3_ = 0.445				
**E.S.R (mL/h)**					
**Median (Min.–Max.)**	32 (12–100)	30 (22–75)	11 (5–33)	K = 138.457 *	<0.001 *
**Sig. bet. grps.**	*p*_1_ = 0.960, *p*_2_ < 0.001 *, *p*_3_ < 0.001 *				
**ALT (U/L)**				K = 16.218 *	<0.001 *
**Median (Min.–Max.)**	14 (8–85)	15 (10–23)	15.5(15–20)		
**Sig. bet. grps.**	*p*_1_ = 0.004 *, *p*_2_ <0.001 *, *p*_3_ = 0.666				
**AST(U/L)**				K = 12.684 *	0.004 *
**Median (Min.–Max.)**	17 (12–70)	17 (12–32)	19.5 (17–22)		
**Sig. bet. grps.**	*p*_1_ = 0.003 *, *p*_2_ = 0.001 *, *p*_3_= 0.756				
**HB (gm/dL)**					
**Mean ± SD.**	10.8 ± 1.3	11.7 ± 1.9	9.7 ± 0.50	F = 47.360 *	<0.001 *
**Sig. bet. grps.**	*p*_1_ < 0.001 *, *p*_2_ < 0.001 *, *p*_3_ < 0.001 *				
**Platelets (×10^3^)**					
**Median (Min.–Max.)**	262 (175–385)	276 (175–332)	236 (175–297)	K = 3.163	0.206
**WBC (×10^3^)**				K = 75.729 *	<0.001 *
**Median (Min.–Max.)**	4.5 (3.4–9.6)	4.6 (3.4–7)	4 (3.4–4.5)		
**Sig. bet. grps.**	*p*_1_ = 0.002 *, *p*_2_ < 0.001 *, *p*_3_ < 0.001 *				
**C.R.P**				K = 25.056 *	<0.001 *
**Median (Min.–Max.)**	12 (5–34)	12 (4–25)	10 (5–20)		
**Sig. bet. grps.**	*p*_1_ = 0.265, *p*_2_ < 0.001 *, *p*_3_ = 0.002 *				
**Creatinine (mg/dL)**				K = 112.959 *	<0.001 *
**Median (Min.–Max.)**	1.2 (1–6.7)	0.9 (0.10–1.90)	0.90 (0.60–1)		
**Sig. bet. grps.**	*p*_1_ < 0.001 *, *p*_2_ < 0.001 *, *p*_3_ = 0.750				
**Urea (mg/dL)**				K = 24.559 *	<0.001 *
**Median (Min.–Max.)**	34 (15–150)	29 (15–100)	34 (27–36)		
**Sig. bet. grps.**	*p*_1_ < 0.001 *, *p*_2_ < 0.001 *, *p*_3_ = 0.418				
**Albumin–creatinine ratio (ACR) (mg/gm)**				K = 132.840 *	<0.001 *
**Median (Min.–Max.)**	90 (34–230)	18 (9–40)	16.5 (4–28)		
**Sig. bet. grps.**	*p*_1_ < 0.001 *, *p*_2_ < 0.001 *, *p*_3_ = 0.104				

χ^2^: chi-squared test; FE: Fisher’s exact test; F: F for ANOVA test, pairwise comparisons between groups were done using Tukey’s post hoc test. K: K for Kruskal–Wallis test, pairwise comparisons between groups were done using Dunn’s post hoc test for multiple comparisons. *p*: *p*-value for comparing between the studied groups. *p*_1_: *p*-value for comparing between patients with nephritis and those without nephritis. *p*_2_: *p*-value for comparing between patients with nephritis and controls. *p*_3_: *p*-value for comparing between patients without nephritis and controls. *: Statistically significant at *p* ≤ 0.05.

**Table 4:** Comparison between the three studied groups according to *MECP2* rs2734647 and *TIRAP* rs8177374.

Gene	SLE with Nephritis (*n* = 63)	SLE without Nephritis (*n* = 47)	Control (*n* = 100)	*p*_1_	OR_1_ (CI. 95%)	*p*_2_	OR_2_ (CI. 95%)	*p*_3_	OR_3_ (CI. 95%)
***MECP2* rs2734647**									
**CC**	31 (49.2%)	34 (72.3%)	81 (81%)		1.000		1.000		1.000
**CT**	22 (34.9%)	12 (25.5%)	16 (16%)	0.109	2.01 (0.86–4.73)	0.001 *	3.593 (1.67–7.73)	0.180	1.787 (0.76–4.18)
**TT**	10 (15.9%)	1 (2.1%)	3 (3%)	0.026 *	10.97 (1.33–90.69)	0.002 *	8.710 (2.25–33.77)	0.844	0.794 (0.08–7.91)
**(CT + TT)**	32 (50.8%)	13 (27.7%)	19 (19%)	0.016 *	2.700 (1.20–6.06)	<0.001 *	4.401 (2.18–8.88)	0.238	1.630 (0.72–3.67)
**Allele**									
**C**	84 (66.7%)	80 (85.1%)	178 (89%)		1.000		1.000		1.000
**T**	42 (33.3%)	14 (14.9%)	22 (11%)	0.002 *	2.857 (1.45–5.63)	<0.001 *	4.045 (2.27–7.21)	0.344	1.416 (0.69–2.90)
***TIRAP* rs8177374**									
**CC**	29 (46%)	35 (74.5%)	82 (82%)		1.000		1.000		1.000
**CT**	30 (47.6%)	11 (23.4%)	16 (16%)	0.006 *	3.292 (1.41–7.69)	<0.001 *	5.302 (2.53–11.11)	0.279	1.611 (0.68–3.82)
**TT**	4 (6.3%)	1 (2.1%)	2 (2%)	0.169	4.828 (0.51–45.62)	0.052	5.655 (0.98–32.52)	0.899	1.171 (0.10–13.34)
**(CT + TT)**	34 (54%)	12 (25.5%)	18 (18%)	0.003 *	3.420 (1.50–7.78)	<0.001 *	5.341 (2.6–10.88)	0.293	1.562 (0.68–3.58)
**Allele**									
**C**	88 (69.8%)	81 (86.2%)	180 (90%)		1.000		1.000		1.000
**T**	38 (30.2%)	13 (13.8%)	20 (10%)	0.005 *	2.691 (1.34–5.41)	<0.001 *	3.886 (2.14–7.07)	0.334	1.444 (0.68–3.04)

OR_1_: Odds ratio for patients with and without nephritis. OR_2_: Odds ratio for patients with nephritis and controls. OR_3_: Odds ratio for patients without nephritis and controls. CI: confidence interval; LL: lower limit; UL: upper limit. *: Statistically significant at *p* ≤ 0.05. *p*_1_: *p* value for comparing between with nephritis and without nephritis. *p*_2_: *p* value for comparing between with nephritis and control. *p*_3_: *p* value for comparing between without nephritis and control.

**Table 5:** Relationships between *MECP2* rs2734647 genotypes and different parameters in SLE patients with nephritis (*n* = 63).

Variables	Mean/Median	*MECP2* rs2734647		Test of Sig.	*p*
		CC (*n* = 31)	CT + TT (*n* = 32)		
**Age (years)**	Mean ± SD.	35.7 ± 9	37.1 ± 9	*t* = 0.611	0.544
		
**Occupation**	Housewife	20 (64.5%)	17 (53.1%)	χ^2^ = 0.843	0.359
	Worker	11 (35.5%)	15 (46.9%)		
**Menstrual history**	Irregular	6 (19.4%)	5 (15.6%)	χ^2^ = 0.152	0.697
	Regular	25 (80.6%)	27 (84.4%)		
**VAS**	Median (Min.–Max.)	30 (10–70)	40 (10–90)	U = 390.0	0.138
**Arthralgia**		16 (51.6%)	18 (56.3%)	χ^2^ = 0.136	0.712
**Arthritis**		5 (16.1%)	7 (21.9%)	χ^2^ = 0.337	0.561
**Normal**		10 (32.3%)	7 (21.9%)	χ^2^ = 0.862	0.353
**Pulse**	Median (Min.–Max.)	75 (65–80)	75 (65–80)	U = 465.0	0.636
**Systolic BP**	Mean ± SD.	113.5 ± 18	117.2 ± 14.4	*t* = 0.887	0.378
**Diastolic BP**	Mean ± SD.	77.1 ± 10.4	77.8 ± 6.1	*t* = 0.332	0.741
**Vitamin A (µg/L)**	Median (Min.–Max.)	15.3 (8.7–41)	18.7 (8.7–41)	U = 459.50	0.614
**Th17 %**	Mean ± SD.	11.9 ± 2.7	11.8 ± 2.8	*t* = 0.052	0.959
**Treg %**	Median (Min.–Max.)	2.5 (1.3–6.3)	3.1 (1.3–6.3)	U = 361.50	0.063
**C3**	Mean ± SD.	57.9 ± 11.3	58.7 ± 9.4	*t* = 0.312	0.756
**C4**	Median (Min.–Max.)	9 (8–20)	10 (8–20)	U = 411.50	0.205
**E.S.R**	Median (Min.–Max.)	32 (12–97)	32 (12–100)	U = 413.50	0.255
**ALT(IU/L)**	Median (Min.–Max.)	14 (8–23)	14 (8–85)	U = 480.50	0.828
**AST(IU/L)**	Median (Min.–Max.)	17 (12–27)	17 (12–70)	U = 471.50	0.730
**HB (gm/dL)**	Mean ± SD.	10.8 ± 1.3	10.9 ± 1.4	*t* = 0.326	0.745
**Platelets (×10^3^)**	Median (Min.–Max.)	276 (175–367)	262 (175–385)	U = 462.0	0.637
**WBC (×10^3^)**	Median (Min.–Max.)	4.5 (3.4–9.6)	4.6 (3.4–9.6)	U = 441.0	0.445
**C.R.P**	Median (Min.–Max.)	12 (5–34)	12 (8–34)	U = 473.50	0.755
**Creatinine (mg/dL)**	Median (Min.–Max.)	1.1 (1–2.3)	1.3 (1.1–6.7)	U = 437.0	0.399
**Urea (mg/dL)**	Median (Min.–Max.)	30 (15–150)	36 (25–55)	U = 428.50	0.347
**Albumin creatinine ratio (mg/g)**	Median (Min.–Max.)	55 (34–140)	100 (44–230)	U = 195.0 *	<0.001 *
**Treatment**	Azathioprine	31 (100%)	32 (100%)	–	–
	Cyclophosphamide	1 (3.2%)	9 (28.1%)	χ^2^ = 7.310 *	^MC^*p* = 0.013 *
	Chloroquine phosphate	18 (58.1%)	8 (25%)	χ^2^ = 7.102 *	0.008 *
	Mycophenolate mofetil	12 (38.7%)	15 (46.9%)	χ^2^ = 0.429	0.513
**SLEDAI**	Median (Min.–Max.)	6 (5–11)	6.5 (5–17)	U = 355.0 *	0.047 *
	Median (Min.–Max.)	100 (10–170)	100 (9–170)		

χ^2^: chi-squared test; MC: Monte Carlo; *t*: Student’s *t*-test; U: Mann–Whitney test. *p*: *p*-value for comparing between the studied groups. *: Statistically significant at *p* ≤ 0.05.

**Table 6:** Relationships between *TIRAP* rs8177374 genotypes and different parameters in patients with nephritis (*n* = 63).

Variables	Mean/ Median	*TIRAP* rs8177374		Test of Sig.	*p*
		CC (*n*= 29)	CT + TT (*n* = 34)		
**Age (years)**	Mean ± SD.	36.6 ± 9.3	36.3 ± 8.7	*t* = 0.143	0.886
**Occupation**	Housewife	18 (62.1%)	19 (55.9%)	χ^2^ = 0.248	0.619
	Worker	11 (37.9%)	15 (44.1%)		
**Menstrual history**	Irregular	5 (17.2%)	6 (17.6%)	χ^2^ = 0.002	0.966
	Regular	24 (82.8%)	28 (82.4%)		
**VAS**	Median (Min.–Max.)	30 (10–70)	35 (10–90)	U = 440.0	0.457
**Arthralgia**		18 (62.1%)	16 (47.1%)	χ^2^ = 1.419	0.233
**Arthritis**		4 (13.8%)	8 (23.5%)	χ^2^ = 0.962	0.327
**Normal**		7 (24.1%)	10 (29.4%)	χ^2^ = 0.221	0.638
**Pulse**	Median (Min.–Max.)	75 (65–80)	75 (65–80)	U = 379.50	0.082
**Systolic BP**	Mean ± SD.	112.1 ± 16.8	118.2 ± 15.5	*t* = 1.517	0.134
**Diastolic BP**	Mean ± SD.	76.9 ± 10	77.9 ± 6.9	*t* = 0.474	0.638
**Vitamin A (ug/L)**	Median (Min.–Max.)	15.3 (8.7–41)	18.7 (8.7–41)	U = 439.50	0.458
**Th17 %**	Mean ± SD.	11.9 ± 2.8	11.8 ± 2.7	*t* = 0.050	0.960
**Treg %**	Median (Min.–Max.)	2.5 (1.3–6.3)	2.9 (1.3–6.3)	U = 377.50	0.109
**C3**	Mean ± SD.	57.6 ± 10.9	58.9 ± 9.9	*t* = 0.494	0.623
**C4**	Median (Min.–Max.)	10 (8–20)	9 (8–20)	U = 455.50	0.573
**E.S.R (mL/h)**	Median (Min.–Max.)	23 (12–97)	33 (20–100)	U = 344.0 *	0.039 *
**ALT (U/L)**	Median (Min.–Max.)	14 (8–25)	14 (8–85)	U = 431.0	0.384
**AST (U/L)**	Median (Min.–Max.)	17 (12–27)	17 (12–70)	U = 477.0	0.821
**HB (gm/dL)**	Mean ± SD.	10.5 ± 1.4	11.1 ± 1.2	*t* = 1.818	0.074
**Platelets (×10^3^)**	Median (Min.–Max.)	262 (175–367)	276 (175–385)	U = 385.50	0.134
**WBC (×10^3^)**	Median (Min.–Max.)	4.5 (3.4–9.6)	4.6 (3.4–9.6)	U = 390.50	0.153
**C.R.P**	Median (Min.–Max.)	12 (5–34)	12 (6–34)	U = 469.0	0.739
**Creatinine (mg/dL)**	Median (Min.–Max.)	1.1 (1–2.3)	1.3 (1.1–6.7)	U = 389.0	0.136
**Urea (mg/dL)**	Median (Min.–Max.)	30 (15–150)	36 (25–55)	U = 478.50	0.840
**Albumin creatinine ratio (ACR) (mg/g)**	Median (Min.–Max.)	60 (34–140)	100 (43–230)	U = 308.50	0.010 *
**Treatment**	Azathioprine	29 (100%)	34 (100%)	–	–
	Cyclophosphamide	1 (3.4%)	9 (26.5%)	χ^2^ = 6.212 *	^FE^*p* = 0.016 *
	Chloroquine phosphate	18 (62.1%)	8 (23.5%)	χ^2^ = 9.591 *	0.002 *
	Mycophenolate mofetil	10 (34.5%)	17 (50%)	χ^2^ = 1.539	0.215
**SLEDAI**	Median (Min.–Max.)	7 (5–11)	6 (5–17)	U = 479.50	0.849
**Anti-ds-DNA titer**	Median (Min.–Max.)	100 (10–170)	100 (9–170)	U = 474.0	0.788

χ^2^: chi-squared test; FE: Fisher’s exact test; *t*: Student’s *t*-test; U: Mann–Whitney test. *p*: *p*-value for comparing between the studied groups. *: Statistically significant at *p* ≤ 0.05.

## Data Availability

All data and materials support published claims and comply with field standards. The datasets generated and/or analyzed during the current study are not publicly available but are available from the corresponding author on reasonable request.

## Supplementary Materials

The following are available online at https://www.mdpi.com/article/10.3390/biom11091378/s1, Figure S1: Figure S1 Allele discrimination plots and sequencing analysis were constructed for *MECP2 and TIRAP*.
