# Supplementary figures and images for: Prognostic Impact of Genetic Variants of MECP2 and TIRAP on Clinical Outcomes of Systemic Lupus Erythematosus with and without Nephritis
Source: Biomolecules. 2021 Sep 18;11(9):1378. doi: 10.3390/biom11091378 (PMC8466489; doi:10.3390/biom11091378)

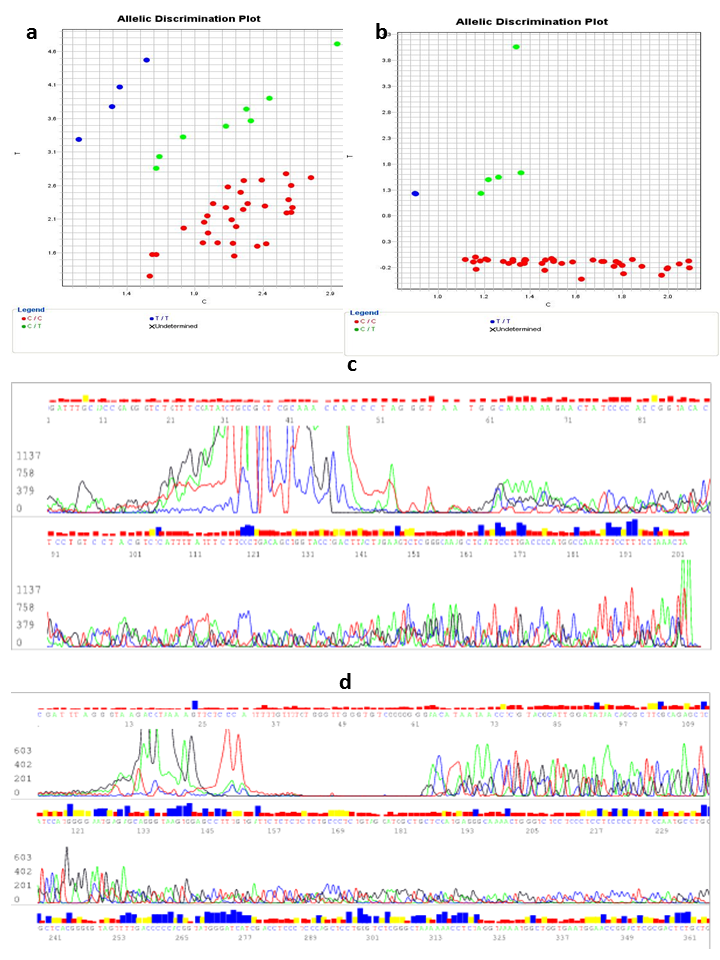

Supplement: Supplementary file 1 [file biomolecules-11-01378-s001.zip › biomolecules-1372875-supplementary.tif]
